# DNA methylation as a potential mediator of the association between indoor air pollution and neurodevelopmental delay in a South African birth cohort

**DOI:** 10.1186/s13148-023-01444-6

**Published:** 2023-02-28

**Authors:** Dakotah Feil, Sarina Abrishamcar, Grace M. Christensen, Aneesa Vanker, Nastassja Koen, Anna Kilanowski, Nadia Hoffman, Catherine J. Wedderburn, Kirsten A. Donald, Michael S. Kobor, Heather J. Zar, Dan J. Stein, Anke Hüls

**Affiliations:** 1 Department of Epidemiology, Rollins School of Public Health, Emory University, 1518 Clifton Road, Atlanta, GA 30322 USA; 2 Department of Paediatrics and Child Health, Red Cross War Memorial Children’s Hospital, SA and SA-MRC Unit on Child and Adolescent Health, University of Cape Town, Cape Town, South Africa; 3 Neuroscience Institute, University of Cape Town, Cape Town, South Africa; 4 Department of Psychiatry and Mental Health, University of Cape Town, Cape Town, South Africa; 5 South African Medical Research Council (SAMRC) Unit on Risk and Resilience in Mental Disorders, University of Cape Town, Cape Town, South Africa; 6 German Research Center for Environmental Health, Institute of Epidemiology, Helmholtz Zentrum München, Neuherberg, Germany; 7 Institute for Medical Information Processing, Biometry, and Epidemiology, Pettenkofer School of Public Health, LMU Munich, Munich, Germany; 8 Division of Metabolic and Nutritional Medicine, Dr. von Hauner Children’s Hospital, University of Munich Medical Center, Munich, Germany; 9 Department of Clinical Research, London School of Hygiene and Tropical Medicine, London, UK; 10 Department of Medical Genetics, University of British Columbia, Vancouver, BC Canada; 11 BC Children’s Hospital Research Institute, Vancouver, BC Canada; 12 Centre for Molecular Medicine and Therapeutics, Vancouver, BC Canada; 13 Gangarosa Department of Environmental Health, Rollins School of Public Health, Emory University, Atlanta, GA USA

**Keywords:** Particulate matter, Neurodevelopment, Epigenetics, Cord blood, Newborn DNA methylation

## Abstract

**Background:**

Exposure to indoor air pollution during pregnancy has been linked to neurodevelopmental delay in toddlers. Epigenetic modification, particularly DNA methylation (DNAm), may explain this link. In this study, we employed three high-dimensional mediation analysis methods (HIMA, DACT, and gHMA) followed by causal mediation analysis to identify differentially methylated CpG sites and genes that mediate the association between indoor air pollution and neurodevelopmental delay. Analyses were performed using data from 142 mother to child pairs from a South African birth cohort, the Drakenstein Child Health Study. DNAm from cord blood was measured using the Infinium MethylationEPIC and HumanMethylation450 arrays. Neurodevelopment was assessed at age 2 years using the Bayley Scores of Infant and Toddler Development, 3rd edition across four domains (cognitive development, general adaptive behavior, language, and motor function). Particulate matter with an aerodynamic diameter of 10 μm or less (PM_10_) was measured inside participants’ homes during the second trimester of pregnancy.

**Results:**

A total of 29 CpG sites and 4 genes (*GOPC*, *RP11-74K11.1*, *DYRK1A*, *RNMT*) were identified as significant mediators of the association between PM_10_ and cognitive neurodevelopment. The estimated proportion mediated (95%-confidence interval) ranged from 0.29 [0.01, 0.86] for cg00694520 to 0.54 [0.11, 1.56] for cg05023582.

**Conclusions:**

Our findings suggest that DNAm may mediate the association between prenatal PM_10_ exposure and cognitive neurodevelopment. *DYRK1A* and several genes that our CpG sites mapped to, including *CNKSR1, IPO13, IFNGR1, LONP2,* and *CDH1,* are associated with biological pathways implicated in cognitive neurodevelopment and three of our identified CpG sites (cg23560546 [*DAPL1*], cg22572779 [*C6orf218*], cg15000966 [*NT5C*]) have been previously associated with fetal brain development. These findings are novel and add to the limited literature investigating the relationship between indoor air pollution, DNAm, and neurodevelopment, particularly in low- and middle-income country settings and non-white populations.

**Supplementary Information:**

The online version contains supplementary material available at 10.1186/s13148-023-01444-6.

## Introduction

The detrimental effects of air pollution on pregnancy outcomes such as low birth weight and respiratory disease in infants are well-known and have been confirmed by many studies over the last several decades [1, 2]. However, there is limited literature on the impact of prenatal air pollution exposure on neurodevelopmental outcomes, and even less work on the biological mechanisms underpinning these associations. A handful of studies have reported significant associations between prenatal air pollution exposure and neurological conditions such as autism spectrum disorder (ASD), attention deficit hyperactivity disorder (ADHD), and more general neurodevelopmental delays [3–7]. However, the bulk of these studies have been conducted in high income country (HIC) contexts and have focused on the effects of outdoor air pollution; therefore, findings may not be wholly generalizable to other settings [8, 9]. It is equally important to address the impact of indoor air pollution, particularly in low- and middle-income country (LMIC) settings where burning fuels such as coal, paraffin, or wood for cooking or heating indoors is common. In such settings, fuel burning can greatly increase indoor air pollution concentration and its impact on neurodevelopment and other health outcomes [5, 10, 11].

Epigenetic modification has long been considered as a key missing link to understanding how gene-environment interactions affect neurodevelopment [12, 13]. As such, careful dissection of the relationship between air pollution, epigenetic modification, and neurological outcomes may allow us to better understand the complex mechanisms underlying the impact of environmental risk factors on neuropsychiatric disorders and neurodevelopment. With the rise of high-throughput genomics, the field of epigenetics has undergone rapid development. Epigenetic modification, specifically DNA methylation (DNAm), has been linked to a number of neuropsychiatric outcomes such as severe neurodevelopmental delay, schizophrenia, ASD, and ADHD [14–20]. DNAm is known to play a key role in embryonic development and has been hypothesized to impact neural stem cell differentiation and maintenance [21], thereby affecting neuropsychiatric outcomes throughout the life course. DNAm is potentially reversible and identification of differentially methylated CpG sites may be useful in multiple contexts, including clinical therapy design and biomarker identification [22].

DNAm levels are altered by a number of environmental exposures such as drugs, nutrition, stress, and air pollution [8, 9, 23, 24]. Data analyzed as part of the Pregnancy and Child Epigenetics (PACE) consortium showed the effects of prenatal exposure to nitrogen dioxide (NO_2_), airborne particulate matter with a diameter of 10 microns or less (PM_10_), and airborne particulate matter with a diameter of 2.5 microns or less (PM_2.5_) on newborn and childhood DNAm [8, 9]. Prenatal exposure to each of these pollutants has been associated with differential DNAm in neonates, highlighting the need for additional research to understand how environment-driven epigenetic changes impact fetal development and downstream health outcomes [8, 9]. While there is evidence of an association between air pollution and DNAm as well as between DNAm and neurodevelopment, few studies have examined the interconnections between them.

To the best of our knowledge, only one study has examined the relationship between prenatal indoor air pollution exposure (PM_10_), DNAm, and neurodevelopment in a mediation analysis [5]. The study was conducted in the Drakenstein Child Health study (DCHS) and investigated deviations of epigenetic gestational age from chronological gestational age (ΔGA) as a potential mediator of the association between indoor air pollution and adverse neurodevelopment. However, this previous study did not find evidence of mediation by ΔGA, leading us to take a more granular approach to understand the role of DNAm as a potential mediator of the association between prenatal indoor air pollution and neurodevelopment [5].

In this study, we aimed to identify any differentially methylated CpG sites and gene regions that mediate the association between prenatal exposure to indoor PM_10_ and neurodevelopment measured at 2 years of age in the DCHS using a combination of high-dimensional mediation analysis methods (HIMA, DACT, and gHMA) and traditional causal mediation analysis [25–27].

## Materials and methods

### Study population

The DCHS is a South African, population-based birth cohort that enrolled 1137 pregnant women with 1143 livebirths from two primary health care clinics in peri-urban communities: Mbekweni and TC Newman. Mothers were recruited during the second trimester of pregnancy and followed throughout the pregnancy. These clinics serve two demographically distinct populations, specifically a majority Black African ancestry community and a majority mixed ancestry community [28]. The DCHS has followed infants from birth until at least 5 years of age [28]. The current study population is composed of 142 mother–child pairs with measures available for cord blood DNA methylation, genotype data, and Bayley Scores of Infant and Toddler Development, 3rd edition (BSID-III) in at least one of the following domains: cognitive development, general adaptive behavior, language, and motor outcomes. Inclusion was also limited to mother–child pairs with measures available for relevant covariates which included principal components (PCs) from genome-wide genotype data, maternal age, maternal smoking, maternal alcohol use, birth weight, sex, and socioeconomic status (SES) (Table 1). Smoking status was determined by maternal urine cotinine levels collected prenatally, while alcohol use was measured via the Alcohol, Smoking and Substance Involvement Screening Test (ASSIST), a tool introduced by the World Health Organization (WHO) and which has shown good validity in LMIC settings [29]. Socioeconomic status was captured as a validated score comprising of four socioeconomic indicators: maternal educational attainment, employment status, household income and assets. [29].

**Table 1:** Study characteristics of the DCHS participants

Characteristic	Participants (*N* = 142)*
Ancestry	
Black	69 (48.6%)
Mixed	73 (51.4%)
Sex	
Female	57 (40.1%)
Alcohol use	
Exposure	28 (19.7%)
Smoking^a^	
Non-smoker	38 (26.8%)
Passive smoker	57 (40.1%)
Active smoker	47 (33.1%)
Maternal HIV status	
HIV +	30 (21.1%)
Birth weight (grams)	3120 (512)
Maternal age (years)	27.20 (5.98)
SES score^b^	0.092 (2.25)
Bayley score—cognitive composite	85.14 (8.65)
Bayley’s score—language composite	84.31 (12.22)
Bayley’s score—motor composite	94.04 (13.74)
Bayley’s score—general adaptive composite	83.72 (13.29)
PM10 concentration (µg/m^3^)	64.6 (96.8)

*The number of participants includes those with data available for all covariates and a composite Bayley’s score in at least one of the relevant domains (Cognitive, General Adaptive, Language, and Motor)

^a^Smoking status was determined by urine cotinine levels (ng/ml), defined as follows: < 10 ng/ml non-smoker, 10–49 ng/ml passive smoker, >  = 500 ng/ml active smoker

^b^SES score description can be found in the supplement section

The DCHS staff obtain written consent from mothers on an annual basis and the study was approved by the Ethics Committee of the Faculty of Health Sciences, University of Cape Town, by Stellenbosch University and the Western Cape Provincial Research committee [28].

### DNA methylation measurements

As described previously [14], DNA was measured from cord blood collected at time of delivery [30]. DNA methylation measures were obtained with both the Illumina Infinium HumanMethylation450 BeadChips (*n* = 156) and the MethylationEPIC BeadChips (*n* = 160). Pre-processing and statistics were done using R 3.5.1 and raw iDat files were imported into Rstudio where intensity values were converted into beta values. The 450 K and EPIC datasets were merged using the minfi R package [31]. Background subtraction, color correction and normalization were performed using the preprocessFunnorm function [32]. Following sample and probe filtering, 273 samples and 409,033 probes remained for downstream analysis. Of these samples, 142 had genotype data, at least one BSID-III score measured at 2 years of age, and data available for all relevant covariates (Table 1). Batch effects were removed using ComBat from the R package sva [33]. Cord blood cell type composition was predicted using the most recent cord blood reference data set [34].

### Neurodevelopment measurements

The BSID-III is a widely used tool for assessing neurodevelopment in children up to 42 months of age. We included scores from across four distinct domains: cognitive development, language skills, motor function, and adaptive behavior [29]. The BSID-III has been validated in LMIC settings and previous research reports its use in the DCHS specifically [29]. As described previously [29], the DCHS assessed neurodevelopment using BSID-III at 2 years of age. The DCHS BSID-III assessment was conducted by a trained professional and incorporates direct observation of the child as well as caregiver input. Composite scores for cognitive, motor, language, and general adaptive behavior domains were scaled to have a mean of 100 and standard deviation of 15 as per standard use of the tool.

### Assessment of indoor air pollution exposure

As described previously [35–37], PM_10_ was measured using a personal air sampling pump (AirChek 52; SKC, Eighty Four, PA, USA), connected to a styrene filter cassette (37 mm cassette blank; SKC) with a gravimetrically pre-weighted filter (PVC filter 37 mm × 5 μm with support pad; SKC) left in the home for 24 h during the 2nd trimester of pregnancy [35, 36]. Filters were weighed after sampling and the National Institute for Occupational Safety and Health method 0600 was used to calculate an average PM_10_ concentration over 24 h [37, 38]. These 24-h average PM_10_ measurements were used for our analyses.

### Statistical analysis

We used different high-dimensional mediation analysis approaches to investigate whether differential DNAm mediates the association between prenatal indoor air pollution exposure and neurodevelopmental delay. Previous research using this subsample of the DCHS has found a significant total effect of PM_10_ on neurodevelopment in the cognitive domain, but not in the BSID-III general adaptive behavior, language, or motor function domains [5]. Therefore, we treated the BSID-III cognitive domain as our primary outcome and the remaining BSID-III neurodevelopment domains were used as secondary outcomes to evaluate consistency of results across domains.

**Fig. 1:**
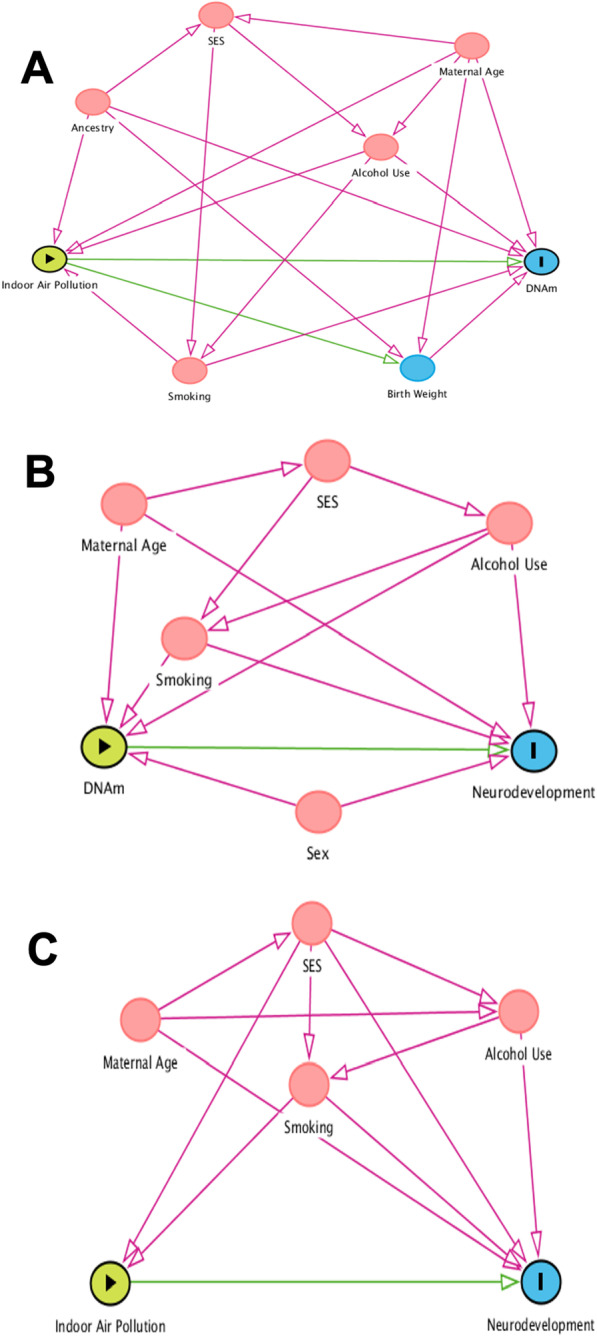
Directed acyclic graphs (DAGs) for exposure-mediator (**A**), mediator-outcome (**B**), and exposure-outcome (**C**) associations. Above are DAGs for each causal pathway relevant to our mediation analysis

Mediation analyses rely on the following three assumptions: (1) no exposure (PM_10_)—mediator (DNAm) confounding, (2) no mediator (DNAm)—outcome (BSID-III Score) confounding and (3) no exposure (PM_10_) -outcome (BSID-III Score) confounding [39]. To fulfill these assumptions to the best of our knowledge, we constructed three directed acyclic graphs (DAGs) to visualize each of these paths (Fig. 1). Confounders were selected based on existing literature [40, 41] and a minimal sufficient adjustment set was identified for each path. Exposure-mediator models were adjusted for SES score, genetic ancestry, and maternal smoking and mediator-outcome models were adjusted for maternal alcohol use, maternal age, SES score, child sex, genetic ancestry, and maternal smoking. We adjusted for genetic ancestry by including the first five genotype PCs to account for population stratification [14]. Models were also adjusted for the first three cell type principal components (PCs), which explained > 90% of cell type heterogeneity [14, 42, 43]. Birth weight as a proxy for gestational age was recognized as another possible mediator of the exposure-mediator association and is a possible mechanism through which prenatal indoor air pollution exposure could impact DNAm; therefore, we did not control for birth weight in our analyses.

To assess the role of DNAm as a potential mediator of the association between prenatal exposure to PM_10_ and neurodevelopment at age 2 years, we employed three well-documented methods for high-dimensional mediation analysis: HIMA (high-dimensional mediation analysis), DACT (divide-aggregate composite null), and gHMA (gene-based high-dimensional mediation analysis) [25–27]. As these methods are novel, there is no consensus as to which is the gold standard [44]. Therefore, we have incorporated an analysis pipeline based on a combination of these methods with traditional causal mediation analysis in order to assess the robustness of results (Additional file 1: Figure S1). It should be noted that all models, used both for high-dimensional mediation analysis and causal mediation analysis, were adjusted for the appropriate confounders as defined above.

HIMA, a high-dimensional mediation analysis method introduced by Zhang et al. [25], employs a dimension reduction technique followed by minimax concave penalty (MCP)-penalized estimation of mediation effects and joint significance testing for mediation effects in order to identify significant mediators. Dimension reduction is performed using the sure independence screening (SIS) method which is built on a correlation learning framework that filters out features that are weakly correlated with the response variable [45]. The HIMA joint significance test rejects the null hypothesis of no mediation only when both the exposure-mediator ($\alpha$) and mediator-outcome effects ($\beta$) are significant [25].

DACT leverages epigenome-wide multiple testing to estimate the proportions of the composite null hypothesis to improve power [26, 44]. A preliminary step for the DACT method is to create two linear models pertaining to each CpG site: the exposure-mediator association ($\alpha$) and the mediator-outcome association ($\beta$). While HIMA uses a screening technique to reduce dimensionality, DACT does not involve a screening step by default. We performed a pre-screen of CpG sites based on their association with the exposure (PM_10_) and the outcome (BSID-III scores of neurodevelopment); only CpG sites with *p* < 0.05 for both associations were included in the downstream analyses (Additional file 1: Figure S1). Given previous findings indicating a negative association between prenatal indoor air pollution exposure and neurodevelopment in the DCHS cohort [5], we chose to additionally filter our sites by only allowing a negative natural indirect effect (NIE) defined by $\alpha *\beta$ (acting in the same direction as the association between indoor air pollution exposure and neurodevelopment). As we chose to pre-screen our CpG sites, we used the Efron correction feature of the DACT package to estimate the proportions of the composite null (Additional file 1: Figure S2) as opposed to Jin and Cai correction which is recommended if performing epigenome-wide mediation effect testing with DACT [26]. Of note, neither of these methods can completely account for correlation between mediators. As such, we calculated Pearson correlation coefficients for all sets of identified CpG sites to better understand if and how these CpG sites were correlated with one another.

The gHMA method was developed by Fang et al. (2020) [27] and focuses on genes as functional units as opposed to individual CpG sites. gHMA is composed of three primary components: (1) linear mediation analysis, (2) nonlinear mediation analysis and (3) an omnibus test of mediation effects. Significance testing results for both the linear and nonlinear mediation analysis steps are combined using the gHMA omnibus (gHMA-O) test. As the true relationship between mediators and outcomes are often not well understood in practice, gHMA-O transforms and combines p-values from the linear and nonlinear analyses in order to construct the gHMA-O test statistic, which is used to assess mediation effects at the gene level [27]. CpG sites were annotated by closest gene using the Bioconductor package hiAnnotator (https://bioconductor.org/packages/release/bioc/html/hiAnnotator.html) and the Ensembl gene predictions (ensGene, version of Apr-06-2014; http://hgdownload.soe.ucsc.edu/goldenPath/.

hg19/database/ensGene.txt.gz) as previously described elsewhere [46].

*p*-values for CpG sites which passed the screening step and were tested using DACT and HIMA were corrected for multiple testing using the Benjamini–Hochberg false discovery rate correction (BH FDR) [47]. Due to the fact that gHMA assesses differentially methylated gene regions as opposed to individual CpG sites, gHMA p-values were FDR corrected for the total number of gene regions tested, rather than the number of distinct CpG sites. HIMA and DACT CpG sites that remained significant at a false discovery rate of 0.05 were then validated via traditional causal mediation analysis using the function *mediate* from the R package *mediation* to obtain estimates of natural indirect effect (NIE), direct effect (DE), total effect (TE), and proportion mediated (PM) [48].

A recent DCHS study identified three CpG sites (cg26971411 [*SPTBN4*], cg00490349 [*intergenic*], cg15660740 [*intergenic*]) associated with neurodevelopment measured by BSID-III [14]. We also examined these CpG sites in our causal mediation analysis step to investigate whether they mediate the association between prenatal PM_10_ exposure and neurodevelopment.

Maternal HIV has been previously linked to neurodevelopment; however, the association between maternal HIV and DNAm is not well understood [49, 50]. Therefore, we also conducted a sensitivity analysis to determine the effect of including maternal HIV as a potential confounder of the mediator-outcome association.

## Results

### Description of study participants

The study sample consisted of 142 mother–child pairs with data available for genotype, cord blood methylation, PM_10_ concentration, scores for one or more BSID-III domains, and all relevant covariates (Table 1). In total, 48.6% of infants were of self-reported Black African ancestry and 51.4% were of self-reported mixed ancestry; 40.1% of infants were female. The mean PM_10_ concentration was 64.5 μg/m^3^ (SD = 96.8 μg/m^3^). Mean composite BSID-III scores were 85.14 (SD = 8.65) for the cognitive domain, 84.31 for the language domain (SD = 12.22), 94.04 for the motor function domain (SD = 13.74), and 83.72 for the general adaptive behavior domain (SD = 13.29). The prevalence of maternal alcohol use was 19.7% and the prevalence of maternal smoking was high, with 40.1% of mothers classed as passive smokers and 33.1% classed as active smokers based on urine cotinine levels. The prevalence of maternal HIV infection was also high with 21.1% of mothers with a confirmed HIV diagnosis.

### CpG-based high-dimensional mediation analysis

**Fig. 2:**
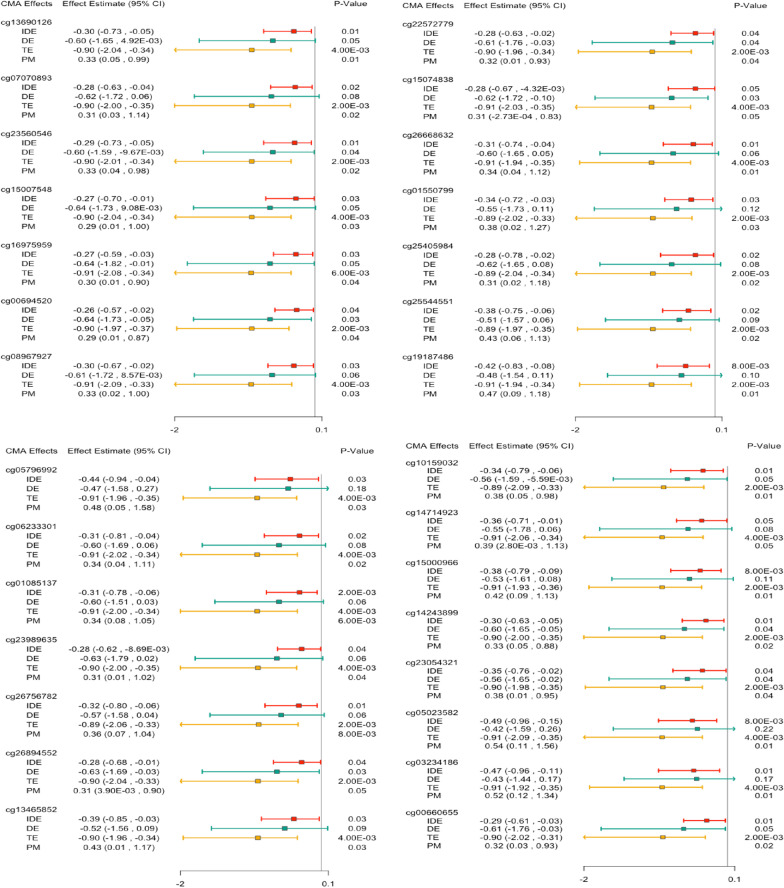
Causal mediation analysis for the association between PM_10_ (exposure) and cognitive neurodevelopment (outcome) using CpG sites identified with high-dimensional mediation analysis methods. This figure presents estimates for indirect effect (IDE), direct effect (DE), total effect (TE), and proportion mediated (PM) for CpG sites with both significant IDE and TE. Significant total effects were found only for the cognitive neurodevelopmental domain (no significant total effects were identified for general adaptive behavior, language, or motor neurodevelopmental domains). IDE, DE, and TE effect estimates have been multiplied by the PM_10_ interquartile range (IQR) (58.78 µg/m^3^) observed in this cohort and thus these effect estimates represent the estimated effects on BSID-III cognitive score per one IQR increase in prenatal PM_10_ exposure

After BH FDR adjustment for multiple testing, DACT identified a total of 123 distinct CpG sites across the cognitive (35 CpG sites, primary outcome), language (45 CpG sites), motor function (13 CpG sites), and general adaptive behavior (39 CpG sites) domains as significant mediators of the association between PM_10_ and neurodevelopment (Additional file 2: Tables S1–S4). A total of 9 CpG sites were shared between at least two domains (Additional file 2: Table S5) and one CpG site (cg26858414 [*CDSN*]) was shared across the language, general adaptive behavior, and motor function domains. These 123 CpG sites were further examined via causal mediation analysis. Results for our primary outcome (cognitive development) are presented here (Table 2; Fig. 2) and results for our secondary outcomes, for which we did not find a total effect of PM_10_ are presented in Additional file 2: Tables S6–S9. The number of probes (DACT and HIMA) and genes (gHMA) present at each step in the analysis pipeline can be found in Additional file 2: Tables S27–S30.

**Table 2:** High-dimensional mediation analysis for the association between PM_10_ (exposure), DNAm (mediator) and neurodevelopment (outcome) using DACT for CpG sites identified via formal causal mediation analysis with a significant indirect effect and total effect

	Chr	Pos	Closest gene	Cognition		Language^b^		General Adaptive^b^	
				Raw *p*-value	FDR	Raw *p*-value	FDR	Raw *p*-value	FDR
cg13690126	1	26,502,524	CNKSR1	1.29E−4	3.30E−3	3.50E−4	5.60E−3	–	–
cg07070893	1	44,412,074	RP11-7O11.3	4.20E−4	7.50E−3	–^a^	–	–	–
cg23560546	2	159,652,019	DAPL1	7.20E−4	0.011	–	–	–	–
cg15007548	2	159,950,276	TANC1	3.70E−3	0.039	–	–	–	–
cg16975959	2	223,184,510	AC010980.2	1.80E−3	0.022	–	–	–	–
cg00694520	2	223,916,687	KCNE4	3.30E−3	0.044	–	–	–	–
cg08967927	5	30,346,164	RP11-136H13.2	4.29E−05	1.40E−3	–	–	–	–
cg22572779	6	10,434,761	RP1-290I10.7	4.5E−4	7.70E−3	–	–	–	–
cg15074838	6	32,406,521	HLA-DRA	2.60E−4	5.50E−3	–	–	–	–
cg26668632	6	137,540,814	IFNGR1	2.90E−4	5.50E−3	–	–	–	–
cg01550799	7	114,561,804	MDFIC	1.80E−3	0.022	–	–	–	–
cg25405984	10	31,074,039	ZNF438	2.90E−3	0.031	–	–	–	–
cg25544551	12	104,531,891	NFYB	1.51E−05	6.40E−4	–	–	–	–
cg19187486	15	74,833,838	ARID3B	3.30E−13	5.87E−11	–	–	–	–
cg05796992	15	101,547,339	LRRK1	4.39E−05	1.40E−3	–	–	–	–
cg06233301	16	1,500,359	CLCN7	2.30E−3	0.027	–	–	–	–
cg01085137	16	48,278,085	ABCC11	1.10E−3	0.015	–	–	–	–
cg23989635	16	68,771,203	CDH1	1.10E−3	0.015	–	–	–	–
cg26756782	16	89,922,218	SPIRE2	5.90E−05	1.60E−3	–	–	–	–
cg26894552	17	5,323,715	RPAIN	2.60E−3	0.029	–	–	–	–
cg13465852	17	27,942,115	CORO6	2.36E−10	2.10E−08	–	–	–	–
cg10159032	17	30,347,989	LRRC37B	2.20E−4	5.30E−3	–	–	–	–
cg14714923	17	43,238,186	HEXIM2	2.34E−07	1.66E−05	–	–	–	–
cg15000966	17	73,128,123	NT5C	1.62E−05	6.4E−4	–	–	–	–
cg14243899	17	74,580,397	ST6GALNAC2	9.00E−4	0.013	–	–	–	–
cg23054321	19	2,289,682	LINGO3	3.36E−07	1.99E−05	–	–	6.97E−09	6.76E−07
cg05023582	19	50,093,541	PRRG2	2.42E−11	2.87E−09	–	–	–	–
cg03234186	19	58,220,657	ZNF551	8.94E−16	3.18E−13	3.05E−26	1.26E−23	–	–
cg00660655	21	46,368,151	FAM207A	7.20E−4	0.011	–	–	–	–

This table includes raw and corrected *p*-values from the DACT method for CpG sites identified across domains restricted to those for which we found with nominally significant indirect effects and total effects (domain-dependent) via causal mediation analysis following high-dimensional mediation analysis (BH FDR <  = 0.05)

^a^Missingness in this table is resultant of our DACT filtering process which is described in more detail in the methods section

35 CpG sites were considered for multiple testing correction in the cognition domain, 45 in the language domain,

39 in the general adaptive behavior domain, and 13 in the motor function domain

^b^No CpG sites with both a significant indirect effect and significant total effect were identified in the language, motor, or general adaptive behavior domains. Two significant CpG sites identified in the cognitive domain overlap with two in the language domain and one in the general adaptive domain and therefore, information for two CpG sites identified with DACT are depicted for the language domain and one for the general adaptive domain alongside the cognition domain

Of the 35 CpG sites identified with DACT for the cognitive domain, 29 demonstrated significant natural indirect effects (NIE), significant estimates for proportion mediated, and significant estimates for total effect (TE). Two of these CpG sites (cg13690126 [*CNKSR1*] and cg03234186 [*ZNF154*]) were also identified via DACT for the language domain (Table 2; Fig. 2). All effect estimates were multiplied by the interquartile range (IQR) of PM_10_ observed in this cohort (58.78 μg/m^3^) and therefore represent estimated effects per one IQR increase in PM_10_. Estimated proportion mediated (95%-confidence interval) ranged from 0.29 [0.014, 0.87] for cg00694520 [*KCNE4*] to 0.54 [0.11, 1.56] for cg05023582 [*PRRG2*]. Cg05023582 also showed the largest NIE estimate (95%-confidence interval) of − 0.49 [− 0.959, − 0.146] per one IQR increase in PM_10_ (Fig. 2).

After correction for multiple testing, HIMA did not identify any CpG sites that significantly mediated the effects of prenatal PM_10_ exposure on neurodevelopment in any domain (Additional file 2: Tables S10–S13). However, prior to multiple testing correction, one CpG site was identified as a significant mediator (cg05796992 [*LRRK1*]); this site was also identified with DACT and demonstrated a significant NIE estimate (95%-confidence interval) of − 0.438 (− 0.942, − 0.0426) per one IQR increase in PM_10_ and an estimated proportion mediated (95%-confidence interval) of 0.484 (0.0468, 1.58) in our causal mediation analysis (Fig. 2, Additional file 2: Tables S6, S14–S15).

We also conducted a sensitivity analysis to examine the effects of adjusting for maternal HIV status. In this sensitivity analysis, 27 of our 29 CpG sites remained significant at a threshold of 0.05 after adjusting for maternal HIV status with only cg16975959 [*intergenic*] and cg15074838 [*HLA-DRA*] losing significance after adjustment (Additional file 2: Tables S16–S19).

Additionally, we tested the mediation effects of three CpG sites that were identified in a recent DCHS study in association with severe neurodevelopmental delay in the cognitive (cg26971411 [*SPTBN4*], cg00490349 [*intergenic*], and cg15660740 [*intergenic*]), language (cg26971411 [*SPTBN4*] & cg00490349 [*intergenic*]), and motor (cg26971411 [*SPTBN4*] & cg00490349 [*intergenic*]) domains using causal mediation analysis [14]. We did not observe significant evidence of mediation in any corresponding BSID-III domain for these CpG sites (Additional file 2: Table S25). This discrepancy may be explained by a lack of association between these three CpG sites and prenatal exposure to PM_10_.

### Gene-based high-dimensional mediation analysis

Differential methylation in four gene regions (*GOPC*, *RP11-74K11.1*, *RNMT*, and *DYRK1A*) was identified as a significant mediator of the association between PM_10_ and cognitive development (Table 3). Each of these differentially methylated gene regions were also identified for the other domains (Table 3). No CpG sites mapping to any of these four genes were identified for the cognitive domain with either of the CpG site-based methods (DACT or HIMA) (Additional file 2: Tables S20-S23).

**Table 3:** Gene-based high-dimensional mediation analysis for the association between PM_10_ (exposure), DNAm (mediator) and neurodevelopment (outcome) using gHMA

Closest gene	CpG count	Cognitive		General adaptive behavior		Language		Motor	
		Raw *p*-value	FDR	Raw *p*-value	FDR	Raw *P*-value	FDR	Raw *P*-value	FDR
*A. Significant mediation for the primary outcome (cognitive domain) and cross-validation across domains*									
GOPC	15	1.19e−09	**4.12e**−**05**	9.73e−03	0.84	8.57e−03	0.98	3.59e−06	**0.04**
RP11-74K11.1	20	1.13e−07	**1.95e**−**03**	1.13e−07	**3.91E**−**3**	1.05e−07	**3.6E**−**3**	5.48e−02	0.89
DYRK1A	19	1.21e−06	**1.39e**−**02**	1.20e−06	**0.02**	1.49e−06	**0.03**	3.78e−03	0.66
RNMT	8	2.86e−06	**2.47e**−**02**	2.87e−06	**0.03**	8.94e−06	0.09	1.22e−02	0.83
*B. Significant mediation for the secondary outcomes (other domains) and cross-validation across domains*									
DCAF13	7	8.92e−05	1.60e−01	8.86e−05	0.17	3.65e−04	0.34	6.52e−06	**0.04**
TNN	13	4.44e−04	3.49e−01	4.40e−04	0.34	1.86e−04	0.31	8.11e−07	**0.03**
TAL1	26	4.61e−03	8.13e−01	1.72e−03	0.58	9.60e−03	1.00	4.06e−06	**0.04**
AC011648.1	3	1.48e−02	1.00e+00	1.49e−02	0.92	1.05e−02	1.00	5.76e−06	**0.04**
SPINK2	5	9.85e−02	1.00e+00	4.14e−01	1.00	4.52e−01	1.00	6.62e−06	**0.04**

Presented associations were significant for at least one neurodevelopmental domain (BH FDR < 0.05). FDR values with bold values indicate significance at a threshold of 0.05

Additionally, we identified five differentially methylated genes (*DCAF13*, *TNN*, *TAL1*, *AC011648.1*, *SPINK2*) as significant mediators for the secondary outcome “motor domain”; however, none of these were found to be significant for the other domains (Table 3).

Lastly, we conducted a sensitivity analysis to examine the effects of adjusting for maternal HIV status on our results. After adjusting for maternal HIV status in our gHMA models, we did not identify any differentially methylated genes as significant mediators using a BH FDR threshold of 0.05 (Additional file 2: Table S24).

## Discussion

This study of 142 mother–child pairs from a low SES population in South Africa found a total of 29 distinct, differentially methylated DNAm probes to significantly mediate the effect of prenatal exposure to PM_10_ on neurodevelopment at age 2 years measured by BSID-III scores. Additionally, we found four differentially methylated gene regions which significantly mediate the effect of prenatal PM_10_ exposure on neurodevelopment using a gene-based high-dimensional mediation analysis technique. To our knowledge, this study is the first to examine differential DNAm at individual probes as potential mediators of the association between prenatal PM_10_ exposure and neurodevelopment.

A number of prior studies have examined the association between prenatal PM_10_ exposure and DNAm as well as the association between DNAm and neurodevelopment [8, 9, 14–20]. While many epigenome-wide association studies (EWAS) have reported differentially methylated CpG sites associated with prenatal air pollution exposure [8, 9, 51–53], we did not identify any overlap between our findings and existing findings. It should be noted that our findings are not entirely comparable as, per the underlying assumptions of mediation analysis, probes must be associated with both PM_10_ exposure and neurodevelopment. Replication is a common problem in EWAS which often lack robust associations at single CpG sites across cohorts [51]. Further research is needed to validate our findings from high-dimensional mediation analysis.

A recent meta-analysis examining epigenome-wide associations between DNAm at birth and childhood cognitive skills synthesizing data from eight pregnancy cohorts within the Pregnancy and Childhood Epigenetics (PACE) consortium (*N* = 3300) did not find substantial evidence that differential cord blood DNAm at individual CpG sites is associated with cognitive skills [19]. We compared our findings to those from several EWAS investigating DNAm and cognitive development examined in the PACE study. However, no overlap was identified between our findings and those of previous studies [19, 54]. Potential explanations for these discrepancies are (1) that we focused on mediation effects of DNAm instead of the direct association between DNAm and cognitive development; (2) differences in the adjustment of multiple testing (we corrected for multiple testing using the BH FDR after an initial pre-filtering process, whereas the PACE meta-analysis used the more conservative Bonferroni threshold on an epigenome-wide scale).

### CpG-based high-dimensional mediation analysis

We identified 29 differentially methylated CpG sites as significant mediators of the association between prenatal PM_10_ exposure and neurodevelopment in the cognitive domain. Of the 29 CpG sites, differential DNAm at 21 of these CpG sites has been associated with age in EWAS examining DNAm trajectories occurring over the course of childhood [55, 56]. Differential methylation at three CpG sites (cg23560546 [*DAPL1*], cg22572779 [*C6orf218*], cg15000966 [*NT5C*]) has been associated with fetal brain development [57]. Differential methylation at one CpG site (cg16975959 [*intergenic*]) has been previously identified as a mediator of the association between maternal smoking and birth weight [58] (Additional file 2: Table S16).

Proportion-mediated estimates for these 29 CpG sites vary, ranging from cg00694520 [*KCNE4*] with 0.29 (0.014, 0.87) to cg05023582 [*PRRG2*] with 0.54 (0.11, 1.56). However, such high PM estimates for each CpG site should be interpreted with caution due to the associated wide confidence intervals, our small sample size, and the fact that causal mediation analysis does not account for correlation between mediators, which we found to be present among these CpG sites (Additional file 1: Figure S3). Several of these CpG sites are located within or adjacent to genes known to influence fetal development and/or neurological outcomes. Herein we discuss CpG sites that map to genes that have been previously linked to neuropsychiatric outcomes.

Cg13690126 is located in *CNKSR1*, a protein-coding gene with low tissue expression specificity. Defects in *CNKSR1* have been linked to syndromic autosomal recessive intellectual disability (ID) [59, 60]. Najmabadi et al. [61] speculate its function as a scaffold protein mediating communication between Ras and Rho GTPase signaling pathways which have in turn been shown to play a role in neurodevelopmental disorders [61, 62]. Cg07070893 is located in a promotor region for Importin 13 (*IPO13*), a gene showing tissue enhanced specificity in the brain and skeletal muscle [59, 63]. *IPO13* is associated with agenesis of the corpus collosum and has been implicated in embryonic stem cell survival. *IPO13* has been proposed as integral to brain development, particularly for the purposes of neural cell-specific cargo trafficking [59, 64, 65].

Cg23560546 is found in an enhancer region of Death Associated Protein Like 1 (*DAPL1*), a protein-coding gene thought to be involved in early stages of epithelial differentiation and/or apoptosis [59]. *DAPL1* has been identified as a significantly differentially methylated region (DMR) in a 2021 study comparing DNAm in peripheral blood cells of toddlers with Down syndrome to neurotypical toddlers [66]. Cg15007548 is located in the gene body of Tetratricopeptide Repeat, Ankyrin Repeat and Coiled-Coil Domain-Containing 1 (*TANC1*) [59]. *TANC1* is a protein-coding gene with low tissue specificity thought to regulate dendritic spines and excitatory synapses [59, 63, 67]. Dendritic spines are integral to synaptic function and loss of function in dendritic spines has been associated with a number of neurological disorders [68]. Cg26668632 is located in a promoter region for *IFNGR1* which belongs to the type II cytokine receptor family and encodes a ligand-binding chain of the gamma interferon receptor (IFN-$\gamma$) [59]. Several studies have found that IFN-$\gamma$ signaling targets play a role in neuronal development and synaptic activity and a recent study [69] suggests that IFN-$\gamma$ signaling is involved in neurodevelopmental disorder etiology [69–71]. Cg23989635 is located in the first exon of Cadherin 1 (*CDH1*), a protein-coding gene that has been implicated in neuronal differentiation and synaptic development in the central nervous system [59, 63, 72–74]. *CDH1* downregulation has been proposed to play a role in congenital neurodevelopmental disorders [75].

Cg0060655 occupies the same position as SNP rs147829886 and is found within an intron on the gene *FAM207A/SLX9* ribosome biogenesis factor*. FAM207A* hypermethylation in umbilical cord tissue has been linked to pre-term birth, which in turn is associated with delayed neurodevelopment [59, 76–78]. Cg23054321 is located in a promotor-associated region proximal to Leucine Rich Repeat and Ig Domain-Containing 3 (*LINGO3*). *LINGO3* is protein-coding gene with tissue enriched in the brain [59, 63]. The *LINGO* gene family has been found to show increased expression as an embryo develops whereas only low levels of these genes are found in adult brains with only *LINGO1* and *LINGO3* being detectable [79]. Epigenetic changes in *LINGO3* have been correlated with depression and a paralog to *LINGO3*, *LINGO1*, acts as a negative regulator of a number of processes key to cognitive function [80]. The genes associated with the remaining CpG sites do not appear to be as well-represented in neuropathological and neurodevelopmental literature. Additional research is needed to elucidate the roles of each of these differentially methylated sites on neurodevelopment.

### Gene-based high-dimensional mediation analysis

gHMA identified four differentially methylated gene regions associated with BSID-III neurodevelopmental scores (*GOPC*, *RP11-74K11.1*, *DYRK1A*, *RNMT*; Table 3). Golgi-Associated PDZ and Coiled-Coil Motif-Containing Protein (*GOPC*) is a protein-coding gene that has been linked to regulation of *GRID2* gene expression which has been shown to impact neurodegeneration [59, 81]. *RP11-74K11.1* is a pseudogene on chromosome 12 which is most highly expressed in the brain, particularly the cerebellum. Few studies have investigated *RP11-74K11.1*, [59, 63, 82]; and therefore additional research is needed to understand its role on neurodevelopment. Dual Specificity Tyrosine Phosphorylation Regulated Kinase 1A (*DYRK1A*) is located on chromosome 21 and encodes a protein kinase. *DYRK1A* has been strongly linked to brain development and function across the life course [63, 83]. Decreased expression of *DYRK1A* has been found in patients with autism spectrum disorder (ASD), while elevated expression has been linked to Down syndrome (DS) [83]. Lastly, RNA Guanine-7 Methyltransferase (*RNMT*) is a protein-coding gene found on chromosome 18; it is known to play a role in RNA-binding and mRNA-methyltransferase activity [59]. The role of *RNMT* in neurodevelopment is not well-documented and further research is needed to better understand if such a link exists.

Although no individual CpG sites located in these gene regions were identified using DACT or HIMA, it is possible that differential methylation on the gene region scale plays a crucial role in neurodevelopment. Probes contained within or proximal to gene regions identified as significant mediators using the gHMA method were eliminated from the DACT analysis pipeline due to lack of significant exposure-mediator and/or mediator-outcome associations (Additional file 2: Tables S16–S19). The discrepancy between differentially methylated gene regions identified with gHMA and gene regions associated with CpG sites identified with DACT may be attributable to interaction effects between proximal, differentially methylated DNAm probes that were not captured via DNAm probe-specific mediation analysis. As both DACT and HIMA do not account for interaction between mediators, these methods may be unable to detect such interacting CpG sites on an individual scale [25, 26].

Our sensitivity analysis, which evaluated the effect of including maternal HIV status as a potential confounder of the mediator-outcome association, showed major differences between gHMA findings with and without adjustment for maternal HIV status. Following adjustment for maternal HIV status, we were unable to identify any differentially methylated gene regions as significant mediators of the association between prenatal PM_10_ exposure and neurodevelopment in any domain (Additional file 2: Table S24). However, in our CpG-based analyses, adjustment for HIV did not greatly alter our findings as both the magnitude and direction of estimated IDE remained consistent (Additional file 2: Table S24). Although maternal HIV status has been linked to neurodevelopmental delay in several studies [49, 50], existing literature is sparse regarding the role of maternal HIV status in DNAm pathways and additional research is needed to better understand if and how maternal HIV status impacts the association between DNAm and neurodevelopment.

### Strengths and limitations

This study has many strengths. It adds to the limited literature dedicated to investigating epigenetic modification and associated outcomes in LMIC settings, particularly related to air pollution exposure. Additionally, the mothers and infants involved in the DCHS are of majority Black African or mixed ancestry, two populations underrepresented in epigenetic and genetic literature at large. To account for population stratification, which may play a role in DNAm variation, our study incorporated genome-wide genotype data which is the preferred approach to account for genetic ancestry. To our knowledge, this is the first study to investigate DNAm as a mediator of the association between prenatal indoor air pollution exposure and neurodevelopment. A unique feature of this study is our use of three different high-dimensional mediation analysis techniques. As no single high-dimensional mediation analysis method has been deemed the gold standard, we employed several methods of high-dimensional mediation analysis in order to compare. Although we did not see good concordance between the methods (Table 2; Additional file 2: Tables S14–S15), we recognize that the probe-specific methods (HIMA and DACT) are differentially powered. A key advantage of the DACT method is that it is better powered than the joint significance test used in HIMA, which tends to be overly conservative [26]. However, additional research is needed to validate these methods and to better understand why findings from these three mediation methods did not demonstrate substantial overlap.

There are several limitations. Our analyses were constrained by a small sample size (*N* = 142), which may have limited the statistical power to detect mediation effects in neurodevelopment across domains. Our sample size may have also limited statistical power to detect underlying associations (total effects) between prenatal PM_10_ exposure and neurodevelopment. Secondly, as DNAm signatures are tissue and cell type specific, our findings are limited in that we investigated DNAm in cord blood and not brain tissue. Although brain tissue DNAm data would have been more appropriate for a study of neurodevelopment, cord blood is far more feasible to collect from living study participants.

## Conclusion

Differential DNAm was found to significantly mediate the association between prenatal exposure to PM_10_ and neurodevelopment as measured by BSID-III at 2 years of age in the DCHS. Twenty-nine differentially methylated CpG sites as well as four differentially methylated gene regions were identified as significant mediators of this association in the DCHS cohort. Due to our small sample size and the general lack of consensus on a gold standard high-dimensional mediation analysis tool in the scientific community, this study should be regarded as a preliminary investigation. Nevertheless, these findings are novel and encourage further research to replicate and expand these results so as to better understand how DNAm and other biological pathways help explain the impact of air pollution exposure on neurodevelopment.

## Supplementary Information

**Additional file 1**: **Figures S1–S3**. Figures outlining the analysis pipeline, DACT goodness-of-fit output, and significant mediator correlations.

**Additional file 2**: **Tables S1–S30**. Tables showcasing HDMA results, CMA results, and findings at each step of the analysis pipeline.

## Data Availability

Not applicable.
