# Supplementary material for: DNA methylation as a potential mediator of the association between indoor air pollution and neurodevelopmental delay in a South African birth cohort
Source: Clin Epigenetics. 2023 Feb 28;15:31. doi: 10.1186/s13148-023-01444-6 (PMC9972733; doi:10.1186/s13148-023-01444-6)
Supplement: Supplementary file 1 — Additional file 1: Figures S1–S3. Figures outlining the analysis pipeline, DACT goodness-of-fit output, and significant mediator correlations. [file 13148_2023_1444_MOESM1_ESM.docx]

**
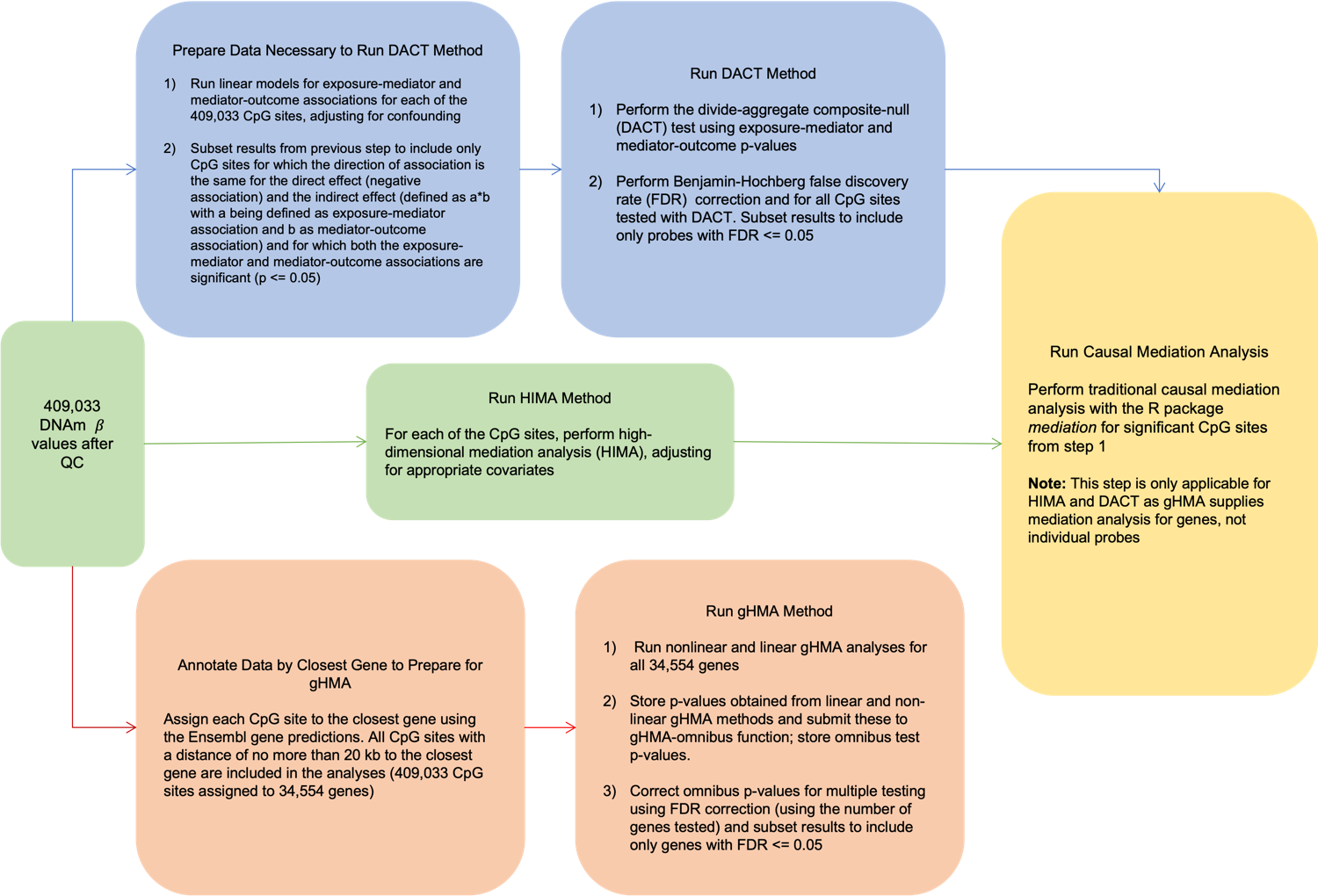
**

**Figure S1. Filtering flowchart.** This figure describes our process of data preparation and filtering for each of the three high dimensional mediation methods used: DACT (blue path), HIMA (green path), and gHMA (orange path).

**
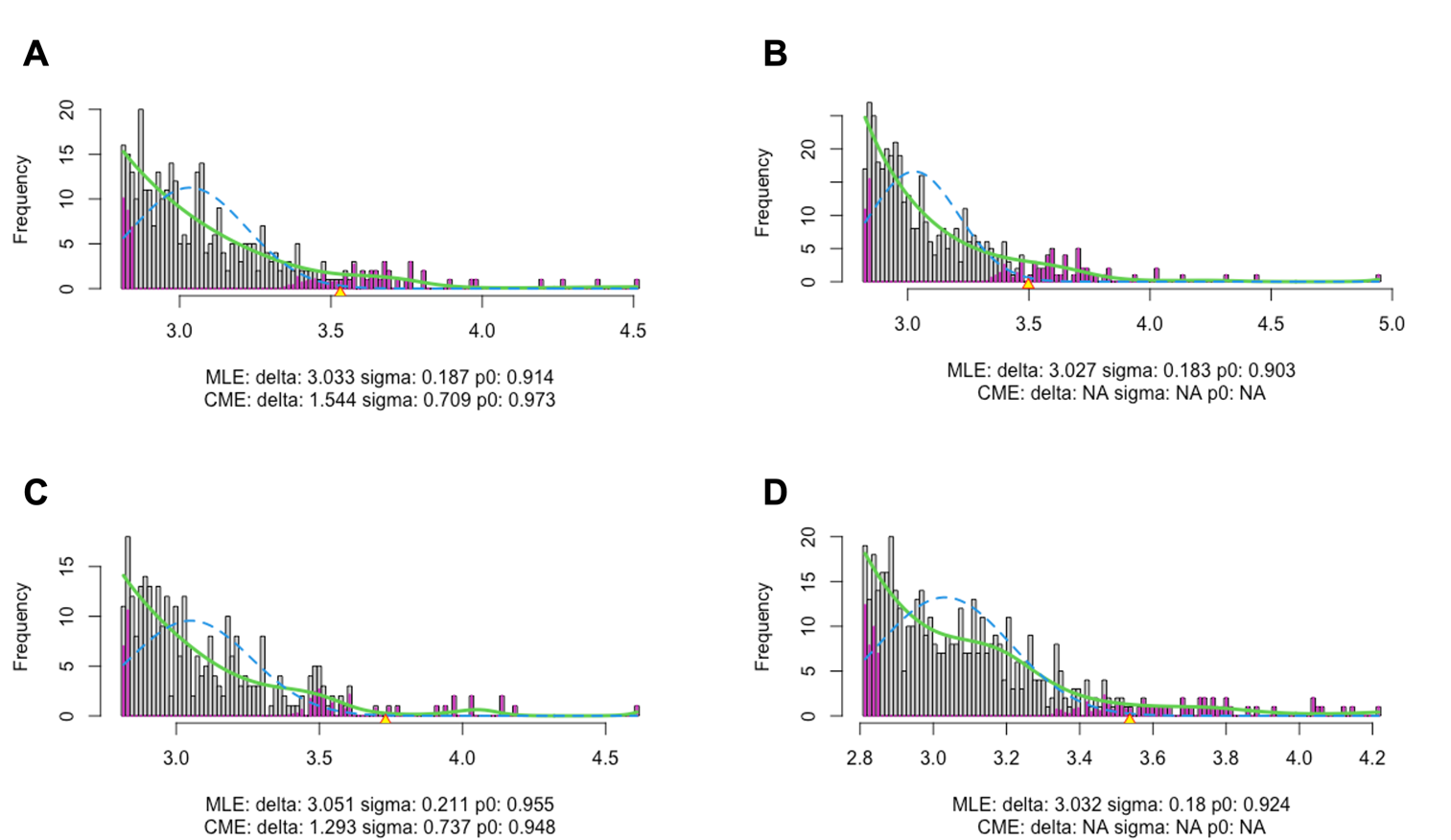
**

**Figure S2. DACT Efron correction goodness-of-fit output from the R package *locfdr*.** R package *locfdr* output for DACT Efron composite null distribution estimation for the following domains: cognitive (1), language (2), motor function (3), and general adaptive behavior (4). The green solid line depicts the spline-based estimate of the mixture density while the blue dashed line is the null sub density as estimated by maximum likelihood (Efron 2015). DACT runs *locfdr* using the default estimation for null sub density, which is estimated by maximum likelihood estimation (MLE).

**
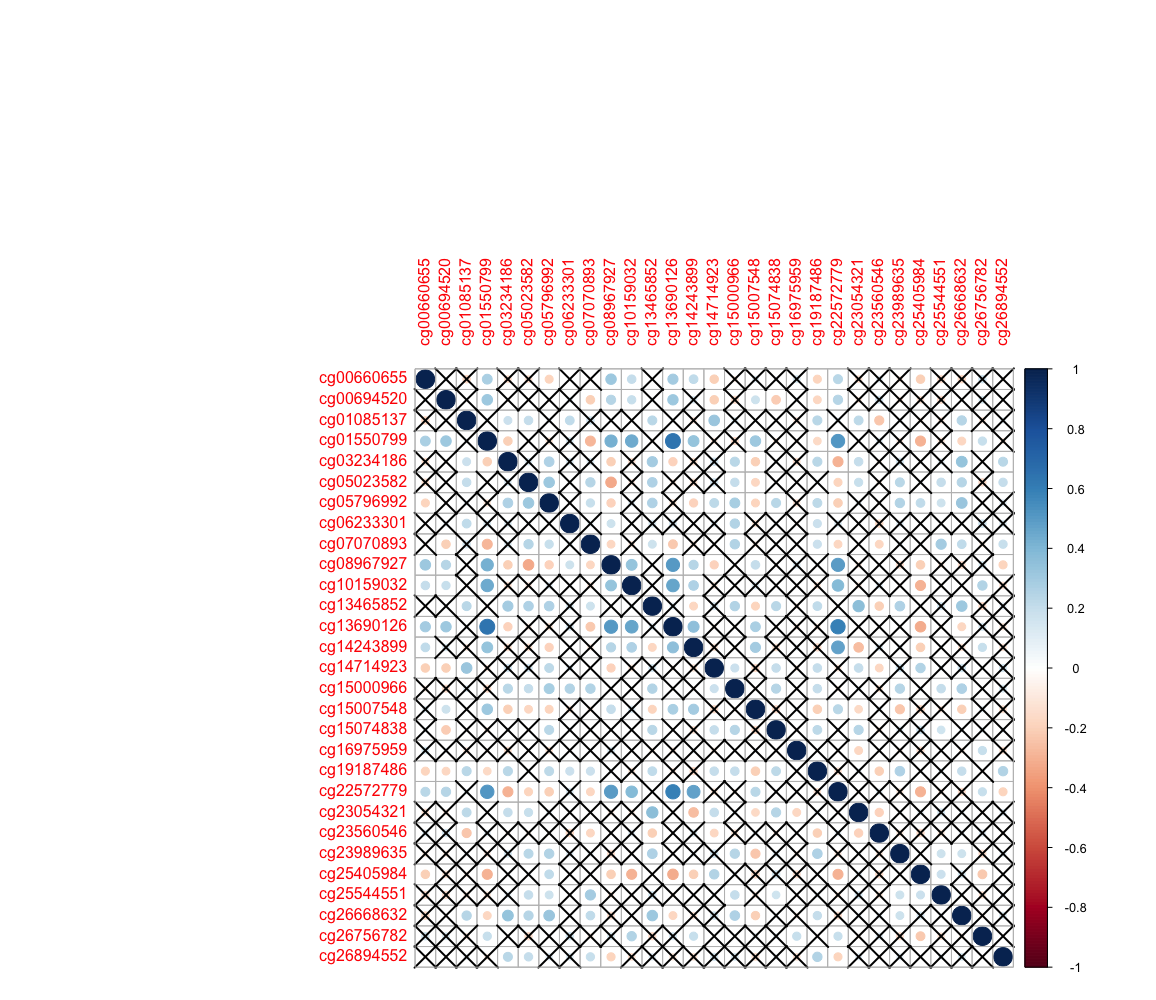
**

**Figure S3. Correlation heatmap for 29 CpG sites identified as significant mediators of the PM10 - cognitive neurodevelopment association.** This heatmap shows Pearson correlation coefficients for each CpG site pair, estimates in red indicate negative coefficients and estimates in blue indicate positive coefficients. The size of the circle is representative of the magnitude of the correlation coefficient. Pairs marked with an X indicate correlations not found to be significant at a threshold of 0.05.
